# A single point mutation in class III ribonucleotide reductase promoter renders *Pseudomonas aeruginosa* PAO1 inefficient for anaerobic growth and infection

**DOI:** 10.1038/s41598-017-14051-2

**Published:** 2017-10-17

**Authors:** Anna Crespo, Joan Gavaldà, Esther Julián, Eduard Torrents

**Affiliations:** 1 Institute for Bioengineering of Catalonia (IBEC), The Barcelona Institute of Science and Technology. Bacterial infections and antimicrobial therapies; Baldiri Reixac 15-21, 08028 Barcelona, Spain; 2 Infectious Diseases Research Laboratory, Infectious Diseases Department, Vall d’Hebron Research Institute VHIR, Hospital Universitari Vall d’Hebron, Barcelona, Spain; 3 Departament de Genètica i de Microbiologia, Facultat de Biociències, Universitat Autònoma de Barcelona, 08193 Bellaterra, Spain

## Abstract

*Pseudomonas aeruginosa* strain PAO1 has become the reference strain in many laboratories. One enzyme that is essential for its cell division is the ribonucleotide reductase (RNR) enzyme that supplies the deoxynucleotides required for DNA synthesis and repair. *P*. *aeruginosa* is one of the few microorganisms that encodes three different RNR classes (Ia, II and III) in its genome, enabling it to grow and adapt to diverse environmental conditions, including during infection. In this work, we demonstrate that a lack of RNR activity induces cell elongation in *P*. *aeruginosa* PAO1. Moreover, RNR gene expression during anaerobiosis differs among *P*. *aeruginosa* strains, with class III highly expressed in *P*. *aeruginosa* clinical isolates relative to the laboratory *P*. *aeruginosa* PAO1 strain. A single point mutation was identified in the *P*. *aeruginosa* PAO1 strain class III RNR promoter region that disrupts its anaerobic transcription by the Dnr regulator. An engineered strain that induces the class III RNR expression allows *P*. *aeruginosa* PAO1 anaerobic growth and increases its virulence to resemble that of clinical strains. Our results demonstrate that *P*. *aeruginosa* PAO1 is adapted to laboratory conditions and is not the best reference strain for anaerobic or infection studies.

## Introduction

*Pseudomonas aeruginosa* is a gram-negative opportunistic pathogen that is responsible for several acute and chronic infections. It presents a significant problem for patients with chronic wounds, cystic fibrosis (CF) and other immunocompromised diseases. The ability of *P*. *aeruginosa* to adapt to diverse environmental conditions and to cause infections relies on its ability to control gene expression in response to environmental stimuli. Additionally, the bacterial mutation rate within the host is a key factor in determining the potential for the bacterial pathogens to genetically adapt to the host immune system and evade drug therapies^1^.

The genome of the wild-type *P*. *aeruginosa* PAO1 strain is relatively large (6.3 Mbp) and contains paralogues of various genes that perform different metabolic activities, thereby allowing for adaption to and exploration of different ecological niches. *P*. *aeruginosa* is one of the few microorganisms that simultaneously encodes three different ribonucleotide reductase (RNR) classes in its genome (class Ia, II and III), allowing it to grow under different specific environmental conditions^2^. RNR enzymes reduce the four different ribonucleotides (NTPs) into their corresponding deoxynucleotides (dNTPs), which are the principal monomers for DNA synthesis and repair; RNR is thus essential for cell division. The genes encoding the three different RNR classes (class I, II and III) share only 10% of their nucleotide composition but perform the same enzymatic activities^3–5^.

The oxygen-dependent class I RNRs consist of two subunits, α and β, in which the α subunit contains the catalytic site and the β subunit contains a metal cofactor. Based on sequence identity and the metal cofactor center, class I RNRs are subdivided into classes Ia, Ib and Ic, which are encoded by the *nrdAB*, *nrdHIEF* and *nrdAB* genes, respectively. Class II RNR enzymes, encoded by the *nrdJ* genes, require 5′-deoxyadenosylcobalamin (AdoCob) or a vitamin B_12_ cofactor for radical generation and do not depend on oxygen for enzymatic activity. Members of the class III RNR, encoded by the *nrdDG* genes, carry a stable but oxygen-sensitive glycyl radical plus an iron-sulfur center that catalyzes the reduction of S-adenosylmethionine to generate its radical. This class can only be active under anaerobic conditions^3,4^

During the infection process, *P*. *aeruginosa* acts under anaerobic conditions^6–8^. However, the *P*. *aeruginosa* strain PAO1 cannot properly grow anaerobically^2,9^. This common reference laboratory strain is a spontaneous chloramphenicol-resistant mutant strain that was isolated in 1954 from a patient wound in Melbourne, Australia (American Type Culture Collection ATCC 15692)^10^ and was found to be equipped with endogenous virulence-suppression mechanisms and to be highly adapted to growth under laboratory conditions. Nevertheless, it is the reference strain in many laboratories for *Pseudomonas* genetics, physiology, identification of virulence factors, metabolic studies and for the identification of specific *Pseudomonas* inhibitors. A debate currently exists concerning whether this laboratory strain is similar to or behaves in the same manner as naturally occurring *P*. *aeruginosa* strains from clinical or natural sources. Other *P*. *aeruginosa* strains that have been more recently isolated, such as *P*. *aeruginosa* PA14, are more adapted to infection conditions and show greater virulence than the PAO1 strain^11,12^. Specifically, *P*. *aeruginosa* PA14 preserves two *P*. *aeruginosa* pathogenicity islands (PAPI-1 and PAPI-2) in its genome^13^. These pathogenic islands occur in several cystic fibrosis (CF) *P*. *aeruginosa* isolates but are absent from *P*. *aeruginosa* PAO1.

We have previously published work on the involvement of the different RNR enzymes during laboratory growth, during biofilm formation and in the virulence of *P*. *aeruginosa* ^2,14,15^. The presence of a class Ia RNR is necessary during aerobic *P*. *aeruginosa* PAO1 growth under laboratory conditions. However, class II and class III RNRs are involved in anaerobic growth, biofilm formation and virulence, although it is difficult to discern the specific importance or role of each RNR class under these specific conditions. Therefore, in this work, we were particularly interested in the specific role of the class III RNR in *P*. *aeruginosa* growth and infection and sought to understand the relative importance of this enzyme in clinical isolates compared to the laboratory strain PAO1. A goal of this study is to clarify the differences in pathogenesis and virulence associated with each *P*. *aeruginosa* strain.

## Results

### Different cell morphologies of *P*. *aeruginosa* strains under anaerobic conditions

**Figure 1:**
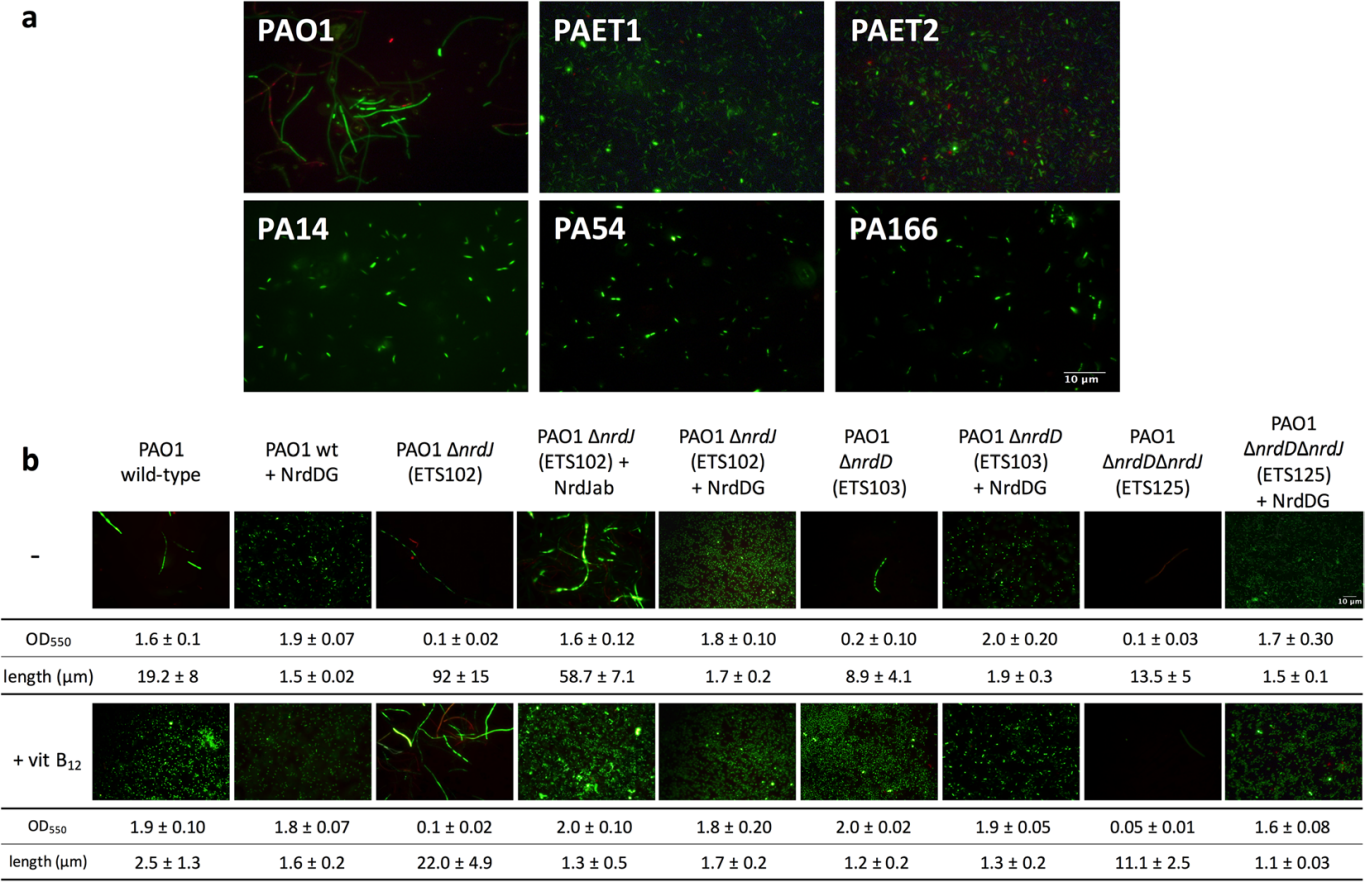
Elongated cell morphology of *P*. *aeruginosa* PAO1 under anaerobic conditions. (**a**) Fluorescence micrographs of *P*. *aeruginosa* PAO1, PAET1, PAET2, PA14, PA54, and PA166 cells grown anaerobically (to an OD_550_ of approximately 0.5) and stained with the LIVE/DEAD assay. (**b**) RNR mutant strains (∆) of *P*. *aeruginosa* PAO1 (ETS102 (∆*nrdJ*), ETS103 (∆*nrdD*), and ETS125 (∆*nrdJ*∆*nrdD*) that contain complementation vectors (pETS159, pBBR1-NrdJab; pETS160, pBBR1-NrdDG; or pETS197, pUCP20T-DG)). Cells were stationary-cultured in the presence or absence of 1 μM vitamin B_12._ Live cells stained with the LIVE/DEAD assay were visualized under a fluorescence microscope (1,000X magnification). The images are representative of three independent experiments with three replicates each. Cell length (mean ± standard deviation) was determined with ImageJ software. Scale bars, 10 μm.

*P*. *aeruginosa* PAO1 cells showed a clear filamentous morphology phenotype (>19 µm long) (Fig. 1a) when grown under anaerobic conditions, indicating impaired DNA replication, as we and other authors have previously suggested^2,9^. Surprisingly, different *P*. *aeruginosa* clinical isolate strains (from cystic fibrosis patients, PAET1 and PAET2; from *P*. *aeruginosa* acute infections, PA54 and PA166; and the laboratory reference strain PA14) showed rod-shaped morphologies (approximately 2 µm long) that were markedly different from that of *P*. *aeruginosa* PAO1 (Fig. 1a). In this case, DNA replication in clinical isolates and PA14 appears unimpaired under anaerobic conditions, in contrast to PAO1.

### Low activity of class III RNR enzymes causes cell elongation in *P*. *aeruginosa* PAO1

Next, we asked why only PAO1 cells were elongated under anaerobic conditions. As expected, PAO1 cells were elongated under anaerobic conditions (average length of 19.2 µm) but were restored to their normal rod shape when the class II RNR cofactor vitamin B_12_ was added to the culture medium (average length of 2.5 µm) (Fig. 1b), indicating that the enzymatic activation of this RNR class restores proper DNA synthesis and replication. No morphological changes were observed in the clinical isolates growing in the absence or presence of vitamin B_12_ (data not shown). When the class II and III RNR genes were mutated (strains ETS102, ET103 and ET125), no anaerobic growth was observed (with an OD of approximately 0.1), and the few visualized cells showed extremely elongated morphologies (from 8.9 to 92 µm long), demonstrating the requirement of anaerobic RNR activity to restore DNA synthesis impairment under anaerobic growth conditions. Indeed, class II RNR activity in the complemented strain (ETS102 + NrdJ) was only restored when the vitamin B_12_ cofactor was present in the culture medium (average length of 1.5 µm). Furthermore, class III RNR complementation (in PAO1 NrdDG^+^, ETS102 NrdDG^+^, ETS103 NrdDG^+^ and ETS125 NrdDG^+^) was sufficient to promote *P*. *aeruginosa* PAO1 growth with wild-type rod-shaped cell morphology (average length of 1.5 µm) (Fig. 1b) without requiring vitamin B_12_. This experiment revealed the relatively low class III RNR expression and activity in *P*. *aeruginosa* PAO1 compared with that in clinical isolates. Our results clearly demonstrated deficient *P*. *aeruginosa* PAO1 DNA replication under anaerobic conditions that was reversed by increasing class III RNR expression levels or gene copy number. Note that constructs in *P*. *aeruginosa* PA14 and PAET1 carrying a mutation in the *nrdD* gene are unable to grow anaerobically indicating class III importance role under these growth conditions (data not shown).

### *nrdD* expression is impaired in *P*. *aeruginosa* PAO1 compared to the clinical isolates

**Figure 2:**
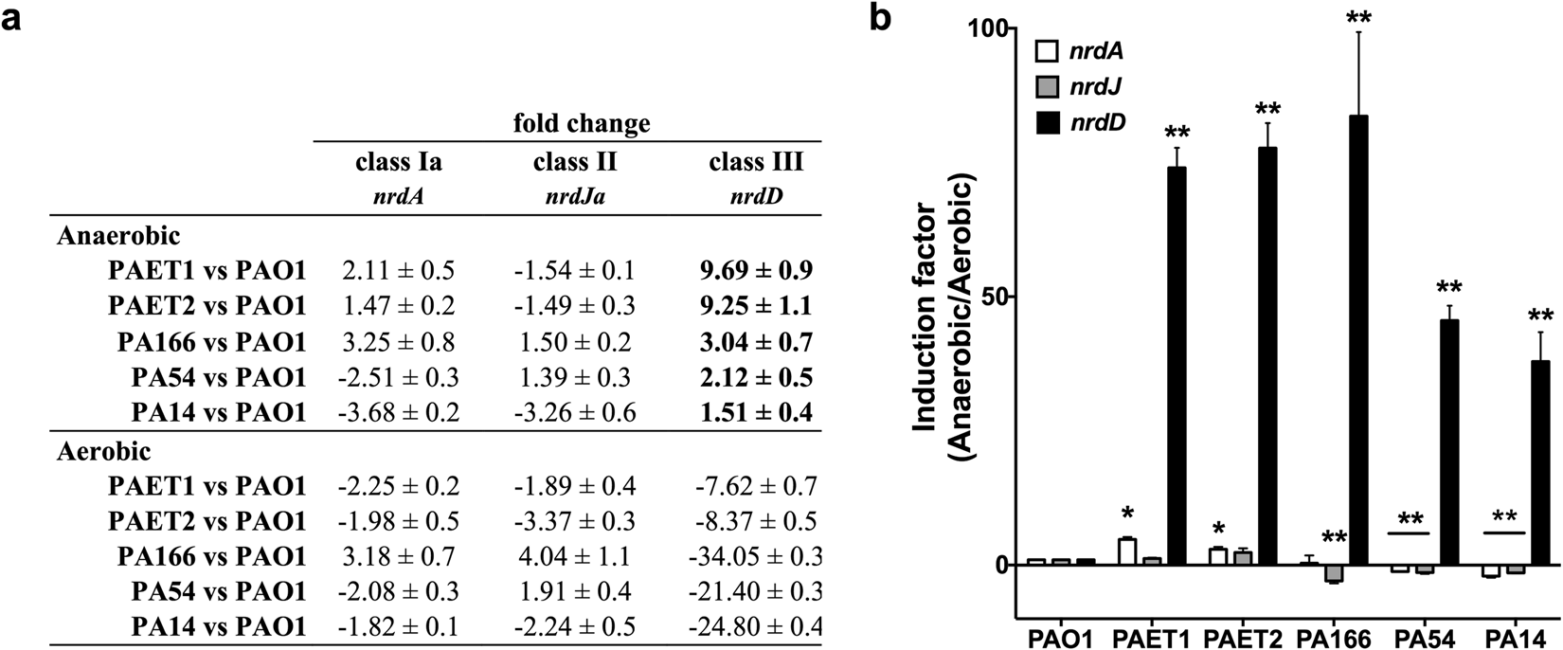
Relative *nrd* gene expression in clinical isolates and laboratory *P*. *aeruginosa* strains. (**a**) Differences in the expression levels of *nrd* genes in PAO1 and in clinical isolate cells grown anaerobically or aerobically at mid-exponential phase (OD_550_ = 0.5). The values in bold represent up-regulated *nrdD* gene. The log fold-change is shown as the mean ± standard deviation of three independent experiments. (**b**) Different RNR (*nrd*) gene expression levels in cells grown anaerobically *versus* cells grown aerobically. The induction expression factor of the *nrdA* (in white), *nrdJ* (in gray) and the *nrdD* (in black) in clinical isolates compared to PAO1 strain. The error bars represent the standard error of the mean. Significantly different from *P*. *aeruginosa* PAO1 in an unpaired *t*-test (*P < 0.05 and **P < 0.0001).

To understand the variation in cell morphology among strains, we analyzed the expression of the genes encoding different RNR classes in *P*. *aeruginosa* laboratory strains (PAO1 and PA14) and in different clinical isolates (PAET1, PAET2, PA166 and PA54) under aerobic or anaerobic growth conditions by qRT-PCR (Fig. 2a).

As previously described, class I RNR (*nrdA*) showed the highest expression level when *P*. *aeruginosa* PAO1 was grown under aerobic conditions, while under anaerobic conditions, class II (*nrdJ*) and III (*nrdD*) RNRs are highly expressed^16^. However, under aerobic conditions, most clinical isolates showed decreased expression of the three *nrd* genes (*nrdA*, *nrdJ* and *nrdD*) relative to PAO1 (Fig. 2a, negative values). The same results were observed under anaerobic conditions, with *nrdA* and *nrdJ* showing reduced expression tendency in the clinical isolates compared with that in the laboratory strain PAO1 (Fig. 2a). However, *nrdD* gene expression was anaerobically upregulated in all the different clinical isolates (9.69 times for PAET1, 9.25 times for PAET2, 3.04 times for PA166, 2.12 times for PA54 and 1.51 times for PA14) compared with that in PAO1, which showed remarkably low expression levels (Fig. 2a).

When we specifically compared the difference in *nrd* expression during anaerobic versus aerobic growth, *nrdD* expression was significantly higher (from 40 to 100 times) in all of the analysed strains than in strain PAO1 (Fig. 2b). This result reveals a marked increase in class III RNR expression in clinical isolates and PA14 strain compared with that in PAO1. Class III RNR activity is responsible for proper anaerobic growth with optimum DNA replication, thereby permitting the rod-shaped morphology observed in Fig. 1.

### A single point mutation renders *P*. *aeruginosa* PAO1 deficient for anaerobic growth

**Figure 3:**
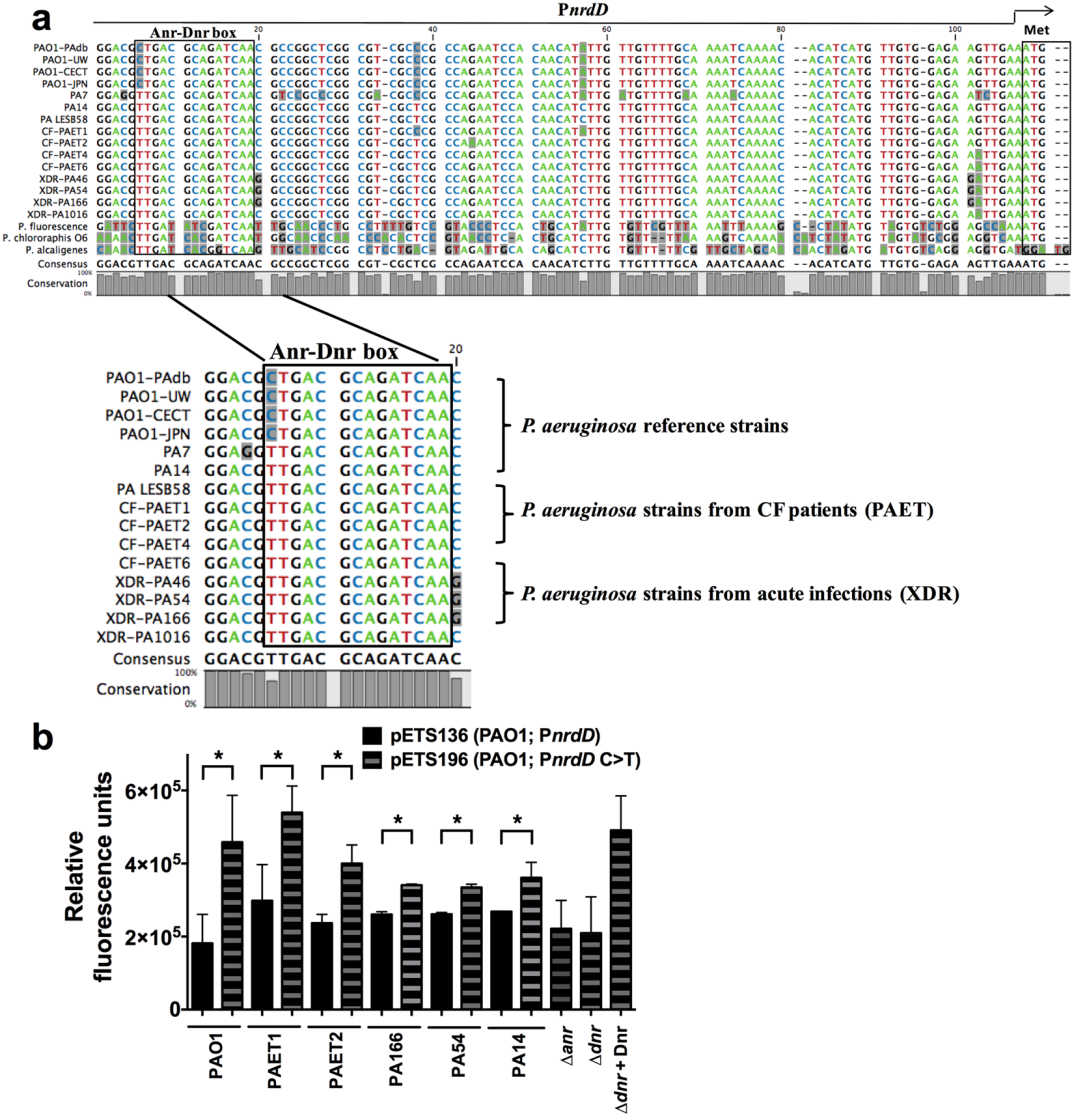
n*rdD* promoter variations in different *P*. *aeruginosa* strains. (**a**) Multiple alignment of the *nrdD* promoter region sequences from different *P*. *aeruginosa* PAO1 strains (PAO1-CECT, PAO1-UW, PAO1-JPN) and from strains isolated from patients with CF (PAET) and with acute infections (extensively drug resistant; XDR). *P*. *aeruginosa* PAO1-PAdb, PA7, PA14 and PA-LESB58 sequences, along with *P*. *fluorescens* and *P*. *alcaligenes* sequences, were obtained from the *Pseudomonas* database. The gray background indicates a mismatch in the sequence. Twenty nucleotides of P*nrdD*, corresponding to the Anr-Dnr binding box that predicted the different *Pseudomonas* strains, are magnified. The percentage conservation is indicated in the bars and grey background indicates a mismatch in the consensus sequence. (**b**) Relative fluorescence units of *nrdD* promoter activity (pETS136-C (P*nrdD* of PAO1) or pETS196-T (P*nrdD* C > T)) in *P*. *aeruginosa* PAO1, PAET1, PAET2, PA166, PA54 and PA14 strains. *P*. *aeruginosa* PAO1 isogenic ∆*anr* and ∆*dnr* mutants were used as controls for Anr/Dnr binding. A plasmid carrying an extra copy of the *dnr* gene (pETS195) was used to complement the ∆*dnr* mutation. Three independent experiments were performed, and the mean ± standard deviation is shown. *, values for pETS196-T (P*nrdD* C > T) significantly differ from those for pETS136 (P*nrdD* of PAO1) in an unpaired t-test (*P* < 0.05).

Low levels of class III RNR (NrdD) activity were clearly responsible for the deficient growth of *P*. *aeruginosa* PAO1 under anaerobic conditions. To explain the different *nrdD* transcription levels of the clinical isolates relative to the laboratory PAO1 strain, we analysed their promoter regions in detail. We sequenced the *nrdD* promoter region from all of the different clinical isolates we tested (PAET1, PAET2, PAET4, PAET6, PA1016, PA166 and PA54) and all available laboratory PAO1 strains (PAO1-CECT, PAO1-UW, PAO1-JPN), and the resulting DNA sequences were compared to other known, sequenced *P*. *aeruginosa* strains (PAO1-PAdb, PA14, PA7 and LESB58) and to other related *Pseudomonas* species (*P*. *fluorescens*, *P*. *chloraphis* and *P*. *alicagenes*) (Fig. 3a). The alignment showed nearly 100% identity among the different *P*. *aeruginosa* strains, while some differences were observed when comparing sequences from other non-related *Pseudomonas* species. By using the Virtual Footprint tool from the PRODORIC database^17^, we identified in all *P*. *aeruginosa* strains a putative Anr/Dnr binding box in this promoter region, located at −98 bp from the translation start site in strain PAO1. Surprisingly, this position showed the only base-pair mismatch we identified between PAO1 (**C**TGACGCAGATCAA) and all the clinical isolates and other laboratory strains such as PA14 (**T**TGACGCAGATCAA) (Fig. 3a).

To verify that this nucleotide mismatch influences promoter activity, we analyzed PAO1 strains carrying *nrdD* transcriptional fusions to GFP with different Dnr transcription factor binding box signatures. We transformed *P*. *aeruginosa* PAO1 cells with plasmids bearing the *nrdD* promoter with single point mutations in the Dnr signature (pETS136-**C**TGACGCAGATCAA or with the clinical-isolate *nrdD* promoter containing the T substitution P*nrdD* (*C* > T)) (pETS196-**T**TGACGCAGATCAA) (see Material and Methods). As shown in Fig. 3b, in all analyzed strains, increased P*nrdD* activity was observed for the pETS196-**T** vector compared with that observed for the vector (pETS136-**C**) carrying the native *P*. *aeruginosa* PAO1 promoter. Moreover, when we measured the P*nrdD* expression of pETS136-C (PAO1 *PnrdD*) in the clinical isolates, we observed a decrease in its expression to the same level observed for the PAO1 strain. Therefore, maximal *nrdD* expression is associated with a promoter bearing the Dnr(T)-box signature (**T**TGACGCAGATCAA), which is typically observed in the naturally occurring *P*. *aeruginosa* strains.

The Anr and Dnr transcription factors share a consensus binding box but activate different specific promoters^18^. Anr activates the transcription of the *dnr* gene, following a regulatory cascade^19^. Thus, to determine which transcription factor is responsible for regulating *nrdD*, we analyzed *nrdD* expression (pETS196-T) in isogenic PAO1 strains carrying ∆*anr* and ∆*dnr* mutations. Complementation of the *dnr* mutation with a plasmid-borne *dnr* gene (pETS195) showed that Dnr specifically induced *nrdD* expression (in pETS196-T with P*nrdD* (C > T)), as we previously observed for the *nrdJ* gene^15^. Notably, *nrdD* was expressed at the same level from the pETS196-T vector in the ∆*anr* and ∆*dnr* mutant strains as from the pETS136-C vector with the PAO1 promoter.

### *nrdD* expression and its role during infection

**Figure 4:**
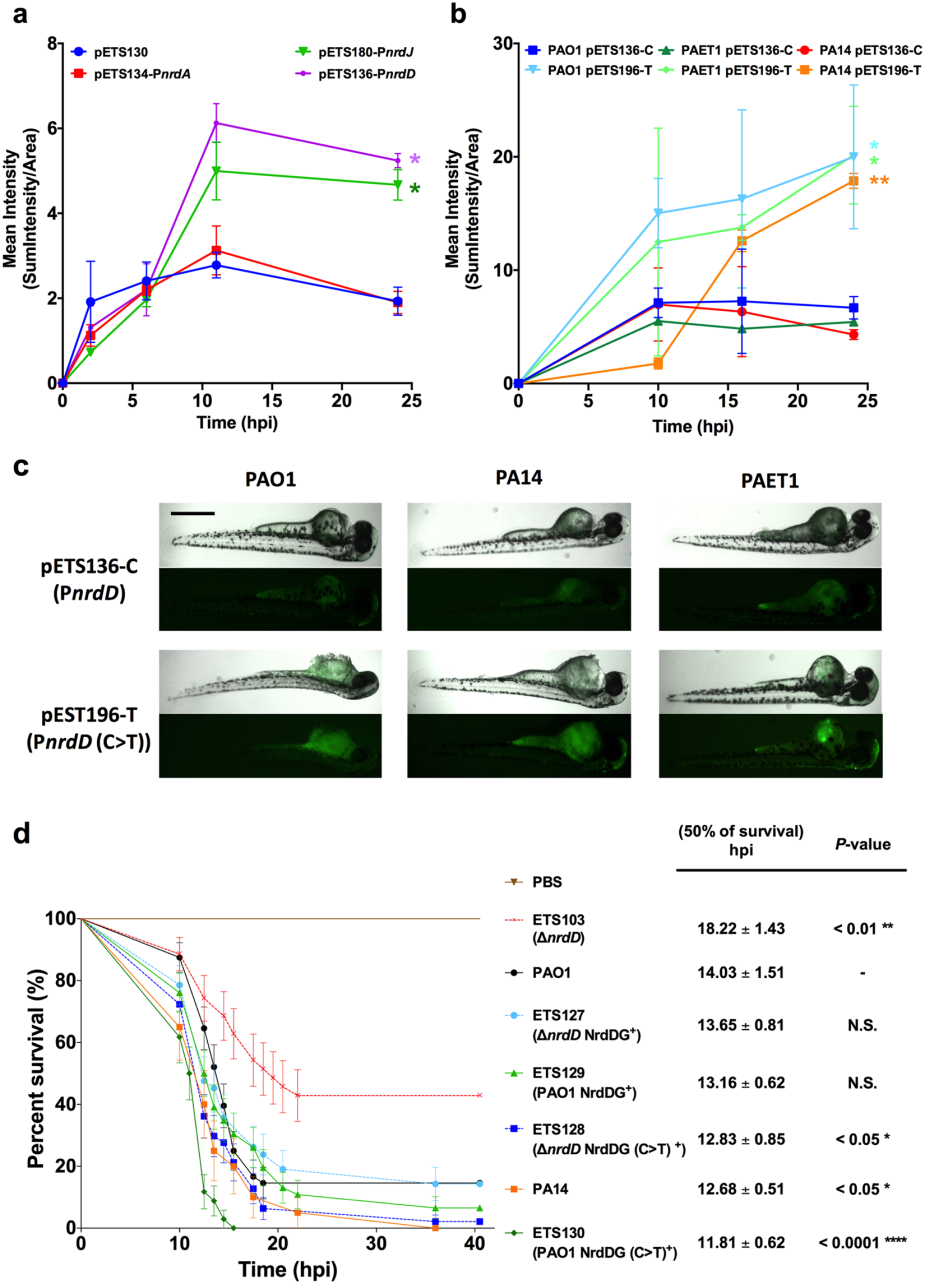
Role of NrdDG in infection. Mean fluorescence intensity values (sum of intensity/area of measurement) in individual embryos infected with (**a**) *P*. *aeruginosa* PAO1 containing the pETS130, pETS134 (*PnrdA*), pETS180 (*PnrdJ*), or pETS136 (*PnrdD*) vectors or (**b**) with different *P*. *aeruginosa* strains (PAO1, PA14 and PAET1) containing the pETS136-C and pETS196-T vectors over 24 h post-infection (hpi). The data represent three independent experiments, with 100 fish analyzed per strain. Statistics were performed to compare strains carrying pETS196-T with strains carrying pETS136-C in an unpaired *t-*test (*P < 0.05 and **P < 0.001). (**c**) Fluorescent and overlaid images of *D*. *rerio* embryos infected with PAO1, PA14 and PAET1 containing the pETS136-C or pETS196-T fluorescent reporter vectors at 16 hpi. Fluorescence was visualized with a fluorescence microscope (Leica MZ16F), quantified with Nikon Nis-element software and processed with ImageJ software. Bars represent 500 μm. (**d**) Kaplan-Meier plots of a survival experiment in *D*. *rerio* infected with different *P*. *aeruginosa* strains (*P*. *aeruginosa* PAO1, PA14, ETS103 (∆*nrdD*), ETS127 (*∆nrdD* PAO1 NrdDG^+^), ETS128 (*∆nrdD* PAO1 NrdDG (C > T)^+^), ETS129 (PAO1 NrdDG^+^) and ETS130 (PAO1 NrdDG (C > T)^+^)). The graph corresponds to a single representative experiment from a total of three independent experiments performed (each using 100 fish per condition). The number of hours’ post-infection (hpi) at which 50% of zebrafish survived are listed with standard deviation. Statistics were performed to compare different strains to *P*. *aeruginosa* PAO1 in a Mantel-Cox test. *P < 0.05, **P *>* 0.001 and ****P < 0.0001; N.S., no significant difference).

We analyzed *nrd* expression *in vivo* by measuring the relative fluorescence during *P*. *aeruginosa* PAO1 infection in the zebrafish (*Danio rerio*) model. The GFP intensity results showed that the *nrdJ* and *nrdD* genes were highly expressed during the course of infection (from 6 until 25 hours post-infection; hpi), with *nrdD* showing the highest expression (Fig. 4a) and following the same expression pattern as observed previously in fly-infection experiments^2^. As shown in Fig. 4b, the expression of *nrdD* during infection was significantly higher in all strains analyzed (PAO1, PA14 and the clinical isolate PAET1) when the promoter contained the C > T modification (pETS196-**T**; P*nrdD* (C > T)) than when it had the native PAO1 sequence (pETS136-**C** (P*nrdD*)).

Finally, we were interested in evaluating whether this single mutation in the *nrdD* promoter, found specifically in *P*. *aeruginosa* PAO1, affects its virulence relative to other strains. We infected zebrafish with wild-type PAO1 strains engineered with the different constructs that showed different *nrdD* expression. We used two *nrdD* merodiploid strains (i.e., with two chromosomal copies), one with the PAO1 wild-type promoter (ETS129; PAO1 NrdDG^+^) and another with the modified P*nrdD* promoter carrying the point mutation of the clinical isolates (ETS130; PAO1 NrdDG (C > T)^+^). We also used strains complemented for the ∆*nrdD* mutation (ETS127, *∆nrdD* PAO1 NrdDG^+^, and ETS128, *∆nrdD* PAO1 NrdDG (C > T)^+^). The *nrdD* expression of each strain was validated and measured using qRT-PCR (Supplementary Fig. S1a), demonstrating that the increased *nrdD* transcription of these strains relative to that from the single copy present in wild-type PAO1 was associated with the rod-shaped morphology found in PA14 and the clinical isolates (Supplementary Fig. S1b).

As expected, the mortality of *D*. *rerio* decreased when we used *P*. *aeruginosa* with an inactivated *nrdD* gene (ETS103; ∆*nrdD*) (18.2 hpi for 50% survival) relative to the mortality after infection with the wild-type strains PAO1 or PA14 (14.03 or 12.7 hpi for 50% survival, respectively) (Fig. 4c). Infection with strains chromosomally complemented for the *nrdD* mutation (strains ETS127, *∆nrdD* PAO1 NrdDG^+^ and ETS128, *∆nrdD* PAO1 NrdDG (C > T)^+^) resulted in survival resembling that of *P*. *aeruginosa* PAO1-infected fish. Furthermore, ETS128 (*∆nrdD* PAO1 NrdDG (C > T)^+^) yielded a similar survival percentage as PA14, but it was significantly increased (*P* < 0.05) over that of PAO1. As previously observed, PA14 showed higher virulence than PAO1^13^. Interestingly, the virulence of the ETS130 (PAO1 NrdDG (C > T)^+^) merodiploid strain was significantly increased (*P* < 0.0001) compared with that of the PAO1 and PA14 wild-type strains due to the elevated *nrdD* expression that increases its anaerobic RNR activity.

## Discussion

*P*. *aeruginosa* is an aerobic bacterium with a versatile metabolism, but its host-infection process is considered to occur under anoxic conditions^6,8,20^. Most *P*. *aeruginosa* patient isolates exhibit strain-dependent differences in response to specific niches and have diverse metabolic activities due to growth in different environments with low nutrients and/or low oxygen gradients^21^. Its large genome and genetic flexibility allow *P*. *aeruginosa* to respond to selective pressure in the host environment by generating mutations in specific genes that allow survival in and adaptation to a variety of infection environments (low oxygen gradients in planktonic and biofilm growth, etc.)^1,22,23^.

Ribonucleotide reductases (specifically, class II and class III RNRs) are described as key enzymes in *P*. *aeruginosa* anaerobic growth and virulence^2,15,24–26^. Previous studies have shown impaired cell division accompanied by an elongated-cell (filamentous) phenotype during *P*. *aeruginosa* PAO1 growth in anaerobic conditions due to the induction of cellular stress by NO levels^25,27^. During denitrification metabolism, nitric oxide (NO) is produced as an intermediate molecule that inhibits the vitamin B_12_ biosynthesis pathway and disrupts class II RNR activity^9,25^. Therefore, addition of vitamin B_12_ (the cofactor of NrdJ) into an anaerobic PAO1 culture prevents cell elongation by increasing NrdJ activity and thus increasing the dNTP synthesis that allows appropriate cell division.

In this study, we showed that *P*. *aeruginosa* clinical isolates have rod-shaped cells under anaerobic conditions, in contrast to filamentous PAO1 cells. We also demonstrated that PAO1 cells had a filamentous phenotype due to a general lack of RNR activity that blocks DNA synthesis in anaerobic conditions (Fig. 1). Under such conditions, the only way for PAO1 cells to grow properly is by the addition of exogenous vitamin B_12_ into the medium to allow fully functional and active class II RNR activity. However, overexpression of the class III RNR system also overcomes the dNTP deficiency and permits the DNA replication required for rod-shaped cell morphology (Fig. 1). Clinical isolates did not show RNR activity deficiency during anaerobic growth, as they show the clear rod-shaped phenotype that is typical of cells with proper cell division.

The reason why clinical isolates show a very different cell morphotype than PAO1 remained unclear. Strikingly, evaluating the expression of different RNR genes under anaerobic conditions showed that *nrdD* expression was elevated, with a concomitant reduction in *nrdJ* (class II RNR) and *nrdA* (class Ia RNR) expression, in the clinical isolates and the PA14 strain relative to PAO1 (Fig. 2). By analyzing this difference (9.69 times for PAET1, 9.25 times for PAET2, 3.04 times for PA166, 2.12 times for PA54 and 1.51 times for PA14), we observed that it was due to a single-base mutation in the promoter region of the *nrdDG* operon that specifically affects the binding of the Dnr transcriptional factor, which is an important regulator of gene induction during anaerobic growth. The PAO1 promoter contains a cytosine (**C**) at the first position of the consensus Dnr box (**C**TGACGCAGATCAA) rather than the thymine (**T**) that is found in the clinical-isolate *nrdD* promoters (Fig. 3a). A substitution of the PAO1 *nrdD* promoter with this specific T nucleotide (pETS196-T, Fig. 3b) returns *nrdD* expression levels to that of the clinical isolates, probably due to more-optimal Dnr transcriptional factor positioning at its binding region. Notably, the affinity of Anr-Dnr for the *arcDABC* promoter and the regulation of the *arcDABC* genes via these transcriptional factors are decreased if the cytosine (C) at the first position of the Anr-Dnr binding box is mutated, implying the same situation as at the *P*. *aeruginosa* PAO1 *nrdD* promoter^28^.

Anr and Dnr are anaerobic transcriptional factors that control most of the genes that are important for anaerobic growth. The global oxygen-sensing regulator is Anr (anaerobic regulator of arginine deiminase and nitrate reductase), which controls *dnr* gene expression. The Dnr regulator is an NO sensor and induces the expression of several genes under anaerobic growth conditions, including during infection^6,29,30^. We have previously demonstrated that Dnr is involved in class II RNR anaerobic expression^15^, and *Escherichia coli* Fnr (an Anr homologue) controls class III RNR expression^31,32^.

Previous work in our laboratory demonstrated that class II RNR from *P*. *aeruginosa* depend on the transcriptional activation by *dnr* ^15^. Here, we also studied the importance of Dnr in the transcriptional regulation of class III RNR (*nrdD*) during biofilm formation by using strains with different Dnr binding affinities. Dnr also induce the expression of class III RNR under biofilm formation (Supplementary Fig. S2a); however, specifically during biofilm formation, class II RNR is the most highly expressed RNR (Supplementary Fig. S2b), as we previously described^15^.

Some studies have found that the class III RNR (NrdDG) is an important protein for bacterial virulence in *E*. *coli* LF82^33^, *Porphyromonas gingivalis* ^34^, *Staphylococcus aureus* ^35,36^, *Streptococcus pneumoniae* ^37^ and *Streptococcus sanguinis* ^38^. For this reason, we attempted to learn whether the single-nucleotide substitution in the *nrdD* promoter region that is specifically found in PAO1 affects its virulence relative to other laboratory strains, such as PA14. PA14 is known to display greater virulence than PAO1^13,39^. Therefore, we analyzed virulence in a *D*. *rerio* zebrafish model of infection using *P*. *aeruginosa* strains with different *nrdD* expression levels (Fig. 4c). As previously observed^2^, mutation of the *nrdD* gene reduces *P*. *aeruginosa* virulence (Fig. 4c). However, the strains that displayed higher *nrdD* expression also showed virulence that was significantly increased relative to wild-type PAO1 and was even higher than that of PA14.

Clearly, in this work, we have seen that *P*. *aeruginosa* uses the class III RNR (NrdDG) for dNTP synthesis, which is important for DNA replication during anaerobic growth and during infection. We specifically identified a single point mutation in the *nrdDG* promoter region that causes PAO1 to grow inefficiently during anaerobic growth and during infection, in contrast to other laboratory *P*. *aeruginosa* strains (e.g., PA14).

Our results indicate that *P*. *aeruginosa* PAO1 is neither appropriate for virulence studies and experiments that require anaerobic metabolism nor in searches for new antimicrobial compounds that involve anaerobic conditions.

## Methods

### Bacterial strains and growth conditions

The bacterial strains and plasmids used in this study are listed in Supplementary Table S1. *E*. *coli* and *P*. *aeruginosa* cells were grown in Luria-Bertani (LB) medium at 37 °C. Anaerobic growth was performed in screw-cap tubes (Hungate Tubes) in LBN medium (LB medium + 10 g/l KNO_3_)^16^. When necessary, antibiotics were added, including ampicillin (Ap) 50 μg/ml, tetracycline (Tc) 12.5 μg/ml and gentamicin (Gm) 10 μg/ml for *E*. *coli* and carbenicillin (Cb) 300 μg/ml, Tc 100 μg/ml and Gm 50 μg/ml for *P*. *aeruginosa*.

### DNA manipulation

Recombinant DNA techniques were performed using standard procedures^16,40^. Plasmids were isolated using the GeneJET Plasmid Miniprep Kit, and DNA fragments were purified using the GeneJET Gel Extraction Kit (Thermo Fisher Scientific, Inc) according to the manufacturer’s instructions. The *P*. *aeruginosa* PAO1 strain that was genetically modified in this work was the PAO1-CECT strain (Supplementary Table S1). DNA was transformed into *P*. *aeruginosa* cells by conjugation or electroporation as previously described^16^.

Using site-directed mutagenesis as previously described^16^ and pETS136 as a template, the identified Anr/Dnr-box at the *nrdD* promoter (see Results section) was mutated at a single nucleotide using the PD-Dnr-T up/PD-Dnr-T low primer pair. The mutated amplicon was cloned into the pETS130 plasmid to generate pETS196-T (P*nrdD* (C > T).

To complement the *nrdDG* deficiency, the complete *nrdDG* genes with their native promoter regions were amplified by PCR using PfuIIDGSacI-up/PfuIIDG-low-BamHI, cloned into pJET1.2, and then further cloned into pUCP20T to generate pUCP20T-DG (pETS197). In addition, the *nrdDG* fragment was also used to construct a merodiploid *P*. *aeruginosa* strain by cloning this DNA fragment into the transposon-containing pBAM-Gm plasmid to generate pBAM-Gm-DG (pETS199). The *nrdDG* (C > T) fragment was generated using PfuIIDGSacI-up/PD-Dnr-T low and PD-Dnr-T up/PfuIIDG-low-BamHI to construct a merodiploid *P*. *aeruginosa* strain with a mutated Anr/Dnr box by cloning this DNA fragment into the transposon-containing pBAM-Gm plasmid to generate pBAM-Gm- DG (C > T) (pETS200). The resulting merodiploid strains were ETS129 (PAO1 NrdDG^+^) and ETS130 (NrdDG (C > T)^+^). Both pBAM-Gm-DG (pETS199) and pBAM-Gm-DG (C > T) (pETS200) were also used for complementation of the ∆*nrdD* strain, thus producing ETS127 (∆*nrdD::Tc* PAO1 NrdDG^+^) and ETS128 (∆*nrdD::Tc* PAO1 NrdDG (C > T)^+^). Constructs were validated by PCR and DNA sequencing.

### RNA extraction, reverse transcription and real-time PCR

Strains of interest were grown to mid-logarithmic phase (OD_550_ = 0.5), and total RNA was extracted using the RNAprotect Bacteria Reagent (Qiagen) and RNeasy Mini Kit (Qiagen) according to the manufacturer’s instructions. To further remove DNA contamination, the eluted RNA was treated with RNAse-free Turbo DNase (Thermo Scientific). The amount of RNA was determined from its 260-nm absorption (NanoDrop spectrophotometer ND-1000, NanoDrop). Reverse transcription-PCR (RT-PCR) was performed using the SuperScript III First-Strand Synthesis System (Thermo Scientific). Quantitative real-time PCR measurements were conducted using SYBR-Green primers (Supplementary Table S2), and detection was performed using an ABI Step One Plus detection system (Applied Biosystems). The *gapA* sequence was used as an internal standard.

### Gene reporter assay for cells grown under anaerobic and biofilm conditions

Strains containing derivatives of the pETS130-GFP plasmid were grown under aerobic conditions to mid-log phase (OD_550_ = 0.5) before different samples were pelleted and inoculated with a needle. The samples were added into Hungate screw-cap tubes containing anaerobic LBN medium for 3 hours to induce anaerobic metabolism. Three independent samples from three independent cultures were collected and fixed with 1X PBS containing 2% formaldehyde. Fluorescence was then measured in 96-well plates using an Infinite 200 Pro fluorescence microplate reader (Tecan). Three measurements were performed for each independent sample.

Gene expression during biofilm formation was determined by growing biofilms as previously described^15^. After 4 days of incubation, the 96-well plate (Nunclon Delta Surface, Thermo Scientific) containing the biofilm was washed to eliminate remaining planktonic cells, and the attached biofilm cells in the wells were fixed with PBS containing 2% formaldehyde. The fluorescence was measured on an Infinite 200 Pro Fluorescence microplate reader.

### Fluorescence microscopic imaging and analysis

Anaerobic overnight cultures of *P*. *aeruginosa* strains were stained using the LIVE/DEAD BacLight viability kit (Thermo Scientific) for 15 minutes at room temperature in the dark. Fluorescent bacteria were visualized with a Nikon E600 microscope (Nikon) coupled with an Olympus DP72 camera. Live cells were visualized in green (SYTO 9 dye), and dead cells were visualized in red (propidium iodide dye). ImageJ software was used for image analysis.

### *nrdD* promoter region sequencing and multiple sequence alignment

The *nrdD* DNA promoter region from different *P*. *aeruginosa* clinical isolates, PA14 and different PAO1 strains from different laboratories (PAO1-CECT from the Spanish Type Culture Collection, PAO1-UW from the Monoil laboratory, and PAO1-JPN from the Japan collection) were amplified by PCR (using the primer pair PnrdD3up/PnrdD-new-low) and then further sequenced using the primer PnrdD3up by the scientific services at the University of Barcelona. We also obtained the *P*. *aeruginosa* PAO1-PAdb, PA14, LESB58, *Pseudomonas fluorescens*, *Pseudomonas chlororaphis* and *Pseudomonas alcaligenes* sequences from the *Pseudomonas* Genome Database^41^. Sequence alignments were performed with CLUSTALW Omega using the default parameters in the CLC Main Workbench software (ver. 6.9.1).

### *P. aeruginosa* infection of *Danio rerio*

The zebrafish (*D*. *rerio*) is a well-established host for studying bacterial virulence mechanisms^42^. Zebrafish embryos from the AB line were a kind gift from Prof. Angel Raya (Center of Regenerative Medicine in Barcelona, Spain). Embryos were kept at 29 °C and staged at 48 hours post–fertilization, and were then dechorionated and anesthetized with 66 μg/mL of ethyl 3-aminobenzoate methanesulfonate (Sigma-Aldrich) prior to infection. Bacterial cells (1,000 cfu) were microinjected into the yolk circulation valley using borosilicate glass capillaries (World Precision Instruments, FL) with a microinjector (TriTech Research, CA). The exact inoculum size in 2–5 nl was determined by viable cell counts from the transfer of cells in the needle to PBS.

For the experiments to determine the expression of the *nrd* genes during fish infection with *P*. *aeruginosa* cells, we used *P*. *aeruginosa* PAO1, PA14 and PAET1 containing the different promoter probe vectors (pETS130, pETS134, pETS136-C, pETS180 and pETS196-T). Fluorescence was measured until 24 hours post-infection (hpi) using a stereo fluorescence microscope (Leica MZ16F) and was analyzed using Nikon Nis-Elements software.

Three independent experiments were performed for the zebrafish survival curve using 100 zebrafish per infecting strain, including PAO1, PA14, ETS103 (∆*nrdD)*, ETS127 (*∆nrdD* PAO1 NrdDG^+^), ETS128 (*∆nrdD* PAO1 NrdDG (C > T)^+^), ETS129 (PAO1 NrdDG^+^) and ETS130 (PAO1 NrdDG (C > T)^+^), over 42 hours post-infection (hpi). The results were plotted in GraphPad Prism 6.0 software using Kaplan-Meier analysis and the log-rank (Mantel-Cox) test to evaluate statistical differences.

### Ethics statement

All zebrafish were raised, maintained and all experiments were performed in accordance to standard protocols, guidelines and regulations described in (zfin.org). Embryos were kept at 29 °C and staged at 48 hours’ post-fertilization. All experiments were conducted following procedures approved by the Ethics Committees on Experimental Animals of the Barcelona Science Park and Barcelona Biomedical Research Park.

### Availability of Data and Materials

All data generated or analyzed during this study are included in this manuscript and it supplementary information files, or is available upon request.

## Electronic supplementary material

Supplementary information
