# Supplementary material for: A single point mutation in class III ribonucleotide reductase promoter renders Pseudomonas aeruginosa PAO1 inefficient for anaerobic growth and infection
Source: Sci Rep. 2017 Oct 17;7:13350. doi: 10.1038/s41598-017-14051-2 (PMC5645315; doi:10.1038/s41598-017-14051-2)
Supplement: Supplementary file 1 — Supplementary information [file 41598_2017_14051_MOESM1_ESM.pdf]

A single point mutation in class III ribonucleotide reductase promoter renders *Pseudomonas aeruginosa* PAO1 inefficient for anaerobic growth and infection

Anna Crespo[1], Joan Gavaldà[2], Esther Julián[3] and Eduard Torrents[1,*]

[1]Institute for Bioengineering of Catalonia (IBEC), The Barcelona Institute of Science and Technology. Bacterial infections and antimicrobial therapies; Baldiri Reixac 15-21, 08028, Barcelona, Spain

[2]Infectious Diseases Research Laboratory, Infectious Diseases Department, Vall d'Hebron Research Institute VHIR, Hospital Universitari Vall d'Hebron, Barcelona, Spain

[3]Departament de Genètica i de Microbiologia, Facultat de Biociències, Universitat Autònoma de Barcelona, 08193 Bellaterra, Spain

*Corresponding author:

etorrents@ibecbarcelona.eu

Supplementary Fig. 1

a)

	Log-fold change		
	nrdA	*nrdJ*	*nrdD*
ETS103 vs PAO1	3.47 ± 0.58	2.64 ± 0.60	-83.96 ± 22.20
ETS127 vs PAO1	-2.23 ± 0.65	-32.18 ± 4.70	1.03 ± 0.20
ETS128 vs PAO1	-1.09 ± 0.25	-10.93 ± 3.40	1.41 ± 0.11
ETS129 vs PAO1	-2.18 ± 0.11	-20.81 ± 3.65	2.01 ± 0.29
ETS130 vs PAO1	-1.33 ± 0.32	-19.01 ± 5.51	2.22 ± 0.16

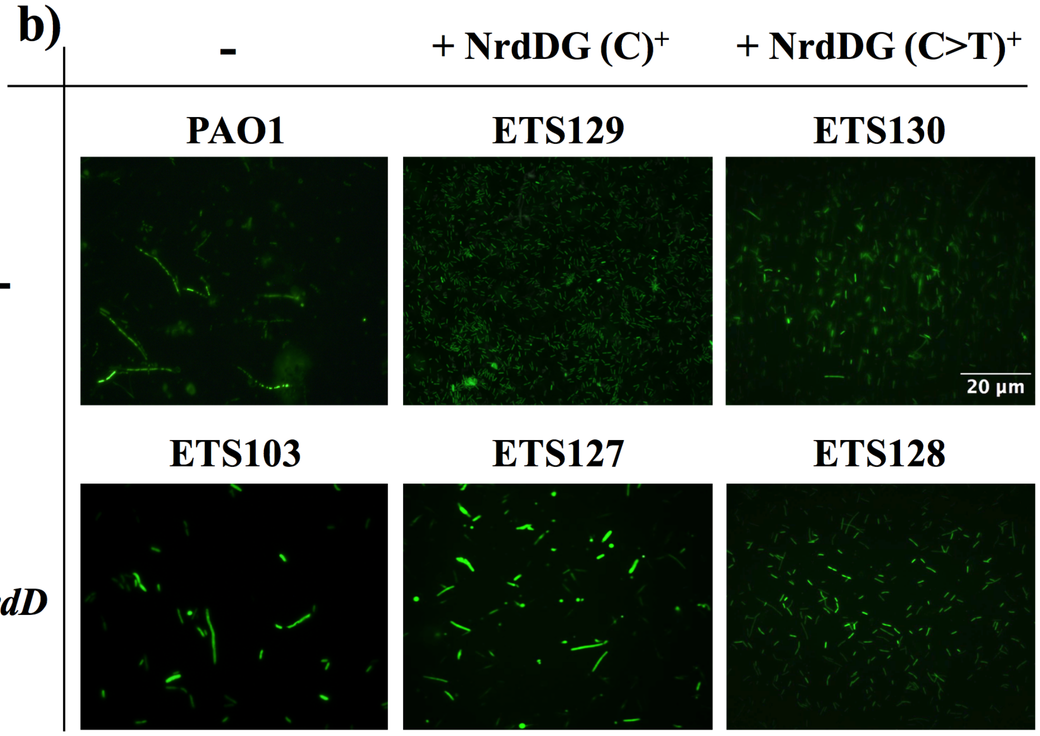

Figure S1. NrdD expression analysis in different *P. aeruginosa* PAO1 strains; microscopic examination of reporter expression and cell morphology. A) Relative *nrd* expression in PAO1, ETS129 (PAO1 NrdDG^{+}), ETS130 (NrdDG (C>T)$^{+}$), ETS127 (Δ*nrdD* PAO1 NrdDG^{+}), ETS128 (Δ*nrdD* PAO1 NrdDG (C>T)$^{+}$) and ETS103 (Δ*nrdD* PAO1) strains as assessed by qRT-PCR. Three independent experiments were performed, and the mean ± standard deviation is shown. **B)** Fluorescence micrographs of an NrdD reporter in *P. aeruginosa* PAO1 strains under anaerobic conditions (at 16 h) using a fluorescence microscope (Leica MZ16F). Cell lengths of wild-type PAO1 (23 µm), ETS127 (Δ*nrdD* PAO1 NrdDG^{+}; 1.6 µm), ETS128 (Δ*nrdD* PAO1 NrdDG (C>T)$^{+}$; 1.4 µm), ETS129 (PAO1 NrdDG^{+}; 1.2 µm), ETS130 (NrdDG (C>T)$^{+}$; 1.3 µm) and ETS103 (Δ*nrdD*; 11 µm)) were measured with ImageJ software. Bars represent 20 µm. The images are representative of three independent experiments.

Supplementary Fig. 2

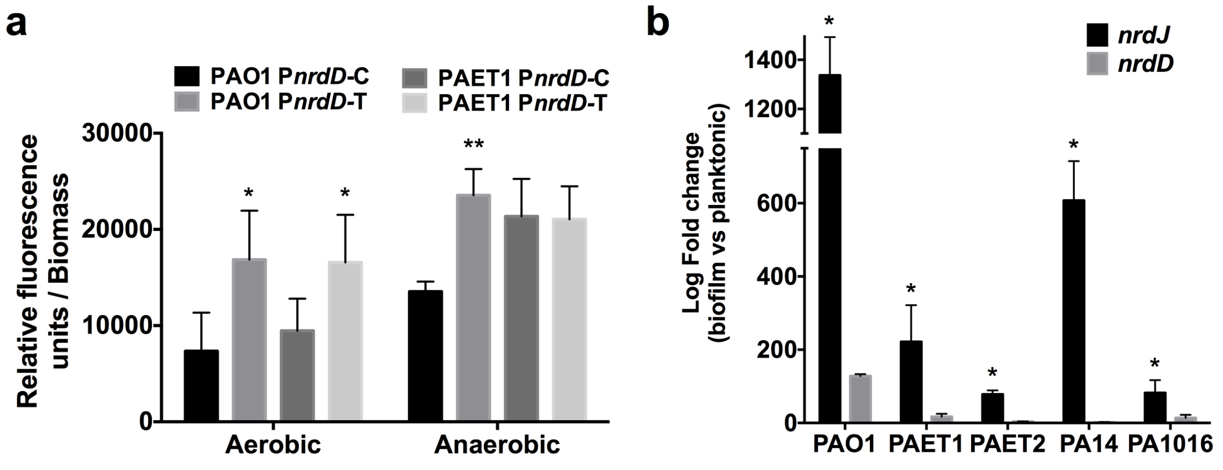

Figure S2. NrdDG during biofilm formation. (**a**) Relative fluorescence of *nrdD* expression from pETS136-C (P*nrdD* of PAO1) and pETS196-T (P*nrdD* (C>T)) after 4 days of PAO1 and PAET1 biofilm growth under aerobic or anaerobic conditions. *, significantly different values for pETS196-T (P*nrdD* C>T) and pETS136 (P*nrdD* of PAO1) in an unpaired t-test ($p<0.05$). (**b**) Relative expression of the *nrdJ* and *nrdD* genes at 4 days of *P. aeruginosa* PAO1, PAET1, PAET2, PA14 or PA1016 biofilm formation compared with planktonic expression by qRT-PCR. The *gapA* gene was used as an internal standard. Three independent experiments were performed, and data with standard deviations were plotted. Significantly different values for *nrdJ* and *nrdD* expression in an unpaired *t*-test (*, $p<0.05$ and **, $p<0.005$).

Supplementary Table S1. Plasmids and strains used in this study.

Strain or plasmid	Description	Source
**Plasmids**		
pBAM-Gm	pBAM derivative carrying Gm^R	This work
pETS130-GFP	Broad host range, promoterless GFP, Gm^R	[1]
pETS134	pETS130 derivative carrying *nrdA* promoter, Gm^R	[1]
pETS136	pETS130 derivative carrying *nrdD* promoter, Gm^R	[1]
pETS159	pBBR1 derivative carrying *nrdJab* operon, Gm^R	[1]
pETS160	pBBR1 derivative carrying *nrdDG* operon, Gm^R	[1]
pETS180	pETS130 derivative carrying *nrdJ* promoter, Gm^R	[2]
pETS195	pUCP20T derivative carrying *dnr* gene, Ap^R	[3]
pETS196	pETS130 derivative carrying *nrdD* promoter (C>T) , Gm^R	This work
pETS197	pUCP20T derivative carrying *nrdDG* gene, Ap^R	This work
pETS199	pBAM-Gm derivative carrying promoter (C) and *nrdDG* genes, Gm^R	This work
pETS200	pBAM-Gm derivative carrying promoter (C>T) and *nrdDG* genes, Gm^R	This work
pJET1.2/blunt	Blunt-end vector, Ap^R	Thermo Scientific
pUCP20T	Broad-host-range vector, Ap^R	[4]
**Strains**		
E. coli		
DH5α	*recA1 endA1 hsdR17 supE44 thi-1 relA1 Δ(lacZYA-argF)U169 deoR Φ80dlacZM15*	Laboratory stock
S17.1 λ*pir*	*recA thi pro hsdR- M+RP4::2-Tc::Mu::Km Tn7 Tpr Smr Xpir*	[5]
P. aeruginosa		
PAO1-CECT	Wild-type (CECT 4122 / ATCC 15692)-Spanish Type Culture Collection	Laboratory stock
PAO1-JPN	Wild-type (ATCC 15692)- Nobuhiki Nomura laboratory	[6]
PAO1-UW	Wild-type (ATCC 15692)- Colin Manoil laboratory	[7]

PA14	Wild-type *P. aeruginosa* PA14	Laboratory stock
PAET1	CF strain isolated from recurrent infection in chronic patient	Laboratory stock
PAET2	CF strain isolated from recurrent infection in chronic patient	Laboratory stock
PAET4	CF strain isolated from recurrent infection in chronic patient	Laboratory stock
PAET6	CF strain isolated from recurrent infection in chronic patient	Laboratory stock
PA1016 (ST175)	Extensively drug resistant strain (XDR)- J. Gavaldà Lab (VHIR)	[8]
PA166 (ST111)	Extensively drug resistant strain (XDR)- J. Gavaldà Lab (VHIR)	[8]
PA54 (ST111)	Extensively drug resistant strain (XDR)- J. Gavaldà Lab (VHIR)	[8]
PW3784 (Δ*anr*)	*P. aeruginosa* PAO1 *anr*::*IS*l*acZ*/hah, Tc^R	[7]
PW1965 (Δ*dnr*)	*P. aeruginosa* PAO1 *dnr*::*IS*l*acZ*/hah, Tc^R	[7]
ETS103 (Δ*nrdD*)	*P. aeruginosa* PAO1 *nrdD*::ΩTc; Tc^R	[1]
ETS102 (Δ*nrdJ*)	*P. aeruginosa* PAO1 *nrdJ*::ΩTc; Tc^R	[1]
ETS125 (Δ*nrdJ*Δ*nrdD*)	*P. aeruginosa* PAO1 *nrdD*::ΩTc; Tc^R ,*nrdJ*::ΩGm; Gm^R	[3]
ETS127 (Δ*nrdD* PAO1+$NrdDG^+$)	*P. aeruginosa* PAO1 *nrdD*::ΩTc, *nrdDG*$^+$ merodiploid, Gm^R Tc^R	This work
ETS128 (Δ*nrdD* PAO1+NrdDG (C>T)$^+$)	*P. aeruginosa* PAO1 *nrdD*::ΩTc; Tc^R, *nrdDG (C>T)*$^+$ merodiploid, Gm^R Tc^R	This work
ETS129 (PAO1+$NrdDG^+$)	*P. aeruginosa* PAO1 *nrdDG*$^+$ merodiploid, Gm^R	This work
ETS130 (PAO1+NrdDG (C>T)$^+$)	*P. aeruginosa* PAO1 *nrdDG (C>T)*$^+$ merodiploid, Gm^R	This work

1 Sjöberg, B. M. & Torrents, E. Shift in ribonucleotide reductase gene expression in *Pseudomonas aeruginosa* during infection. *Infect Immun* **79**, 2663-2669, doi:10.1128/IAI.01212-10 (2011).

2 Crespo, A., Pedraz, L. & Torrents, E. Function of the *Pseudomonas aeruginosa* NrdR Transcription Factor: Global Transcriptomic Analysis and Its Role on Ribonucleotide Reductase Gene Expression. *PloS one* **10**, e0123571, doi:10.1371/journal.pone.0123571 (2015).

3 Crespo, A., Pedraz, L., Astola, J. & Torrents, E. *Pseudomonas aeruginosa* Exhibits Deficient Biofilm Formation in the Absence of Class II and III Ribonucleotide Reductases Due to Hindered Anaerobic Growth. *Frontiers in microbiology* **7**, 688, doi:10.3389/fmicb.2016.00688 (2016).

4 West, S. E., Schweizer, H. P., Dall, C., Sample, A. K. & Runyen-Janecky, L. J. Construction of improved *Escherichia-Pseudomonas* shuttle vectors derived from pUC18/19 and sequence of the region required for their replication in *Pseudomonas aeruginosa*. *Gene* **148**, 81-86, doi:0378-1119(94)90237-2 [pii] (1994).
5 de Lorenzo, V., Cases, I., Herrero, M. & Timmis, K. N. Early and late responses of TOL promoters to pathway inducers: identification of postexponential promoters in *Pseudomonas putida* with lacZ-tet bicistronic reporters. *J Bacteriol* **175**, 6902-6907 (1993).
6 Toyofuku, M. *et al.* Quorum sensing regulates denitrification in *Pseudomonas aeruginosa* PAO1. *J Bacteriol* **189**, 4969-4972, doi:10.1128/JB.00289-07 (2007).
7 Jacobs, M. A. *et al.* Comprehensive transposon mutant library of *Pseudomonas aeruginosa*. *Proc Natl Acad Sci U S A* **100**, 14339-14344, doi:10.1073/pnas.2036282100 (2003).
8 Mulet, X. *et al.* Biological markers of *Pseudomonas aeruginosa* epidemic high-risk clones. *Antimicrob Agents Chemother* **57**, 5527-5535, doi:10.1128/AAC.01481-13 (2013).

Supplementary Table S2. Primers and probes used in this study.

Name	Sequence (5'→3')	Application
greenQRT_PAO-gapA_rv	GGTCATCAGGCCGTGCTC	qRT-PCR
greenQRT_PAO-gapA_fw	CCTCCCATCGGATCGTCTC	qRT-PCR
gapTaqM-low	GAGGTTCTGGTCGTTGGT	cDNA
greenQRT_PAO-nrdA_rv	TGTGGATGAAGTAGCGGTCG	qRT-PCR
greenQRT_PAO-nrdA_fw	ACCTGGAGAAACTGGGCAAG	qRT-PCR
nrdATaqM2-low	TGTTCATGTCGTGGGTACG	cDNA
greenQRT_PAO-nrdD_rv	GGGTGATGTTGTAGGTCGGG	qRT-PCR
greenQRT_PAO-nrdD_fw	AGATGGACCTGATCAACCGC	qRT-PCR
nrdDTaqM2-low	CCGAGTTGAGGAAGTTCTGG	cDNA
greenQRT_PAO-nrdJ_rv	TCCACCGCCTGCATGAAC	qRT-PCR
greenQRT_PAO-nrdJ_fw	CGAATTCATCCGCGCCAAG	qRT-PCR
nrdJTaqM2-low	GTAAACACCCGCACCACTTC	cDNA
pBAM-low	GGAACACTTAACGGCTGACAT	Cloning
pBAM-up	ACGAACCGAACAGGCTTATG	Cloning
PD-Dnr-T low	GATCTGCGTCAACGTCCGGC	Cloning
PD-Dnr-T up	GCCGGACGTTGACGCAGATC	Cloning
PfuIIDG-low-BamHI	AAGGATCCTGAGTCTTGTGAAGGACAGGCC	Cloning
PfuIIDGSacI-up	AAGAGCTCTGGACAACTACGTCGTCTTCGC	Cloning
pJET-rev	AAGAACATCGATTTTCCATGGCAG	Check-Cloning
pJET-up	CGACTCACTATAGGGAGAGCGGC	Check-Cloning
PnrdD new-low	AATCGATCAGGGTGGCCGGCCAGGTAG	Cloning
PnrdD-up	AGGATCCGAATTCGCCCGCCTCGCCCAGG	Cloning
PnrdD3-up	TGCTCGAACGCTTCCCGGCGGC	Sequencing

pUCP20T-low	TCCGGCTCGTATGTTGTGTG	Cloning
pUCP20T-up	CCTCTTCGCTATTACGCCAG	Cloning
